# Endocytic Pathway of Feline Coronavirus for Cell Entry: Differences in Serotype-Dependent Viral Entry Pathway

**DOI:** 10.3390/pathogens8040300

**Published:** 2019-12-16

**Authors:** Tomomi Takano, Yumeho Wakayama, Tomoyoshi Doki

**Affiliations:** Laboratory of Veterinary Infectious Disease, School of Veterinary Medicine, Kitasato University, Towada, Aomori 034-8628, Japan; vm14140h@st.kitasato-u.ac.jp (Y.W.); doki@vmas.kitasato-u.ac.jp (T.D.)

**Keywords:** feline coronavirus, serotype, feline infectious peritonitis, late endosome, macrophage

## Abstract

Feline coronavirus (FCoV) is a pathogen causing a lethal infectious disease in cats, feline infectious peritonitis. It has two serotypes (type I FCoV and type II FCoV). According to our previous study, type I FCoV infection is inhibited by compounds inducing intracellular cholesterol accumulation, whereas type II FCoV infection is not inhibited. Intracellular cholesterol accumulation was reported to disrupt late endosome function. Based on these findings, types I and II FCoV are considered to enter the cytosol through late and early endosomes, respectively. We investigated whether the antiviral activities of a late endosome trafficking inhibitor and cholesterol-accumulating agents are different between the FCoV serotypes. The late endosome trafficking inhibitor did not inhibit type II FCoV infection, but it inhibited type I FCoV infection. Type I FCoV infection was inhibited by cholesterol-accumulating triazoles, but not by non-cholesterol-accumulating triazoles. These phenomena were observed in both feline cell lines and feline primary macrophages. This study provides additional information on the differences in intracellular reproductive cycle between type I and type II FCoV.

## 1. Introduction

Feline coronavirus (FCoV) is a positive-sense RNA virus in the species Alphacoronavirus 1 genus *Alphacoronaivirus*, of the subfamily *Coronavirinae*, in the family *Coronaviridae* [1]. It is divided into two serotypes (types I and II) based on the amino acid sequence of the spike (S) protein [2]. Type I FCoV is the dominant serotype (80–90%) in Europe and Asia [3,4,5]. FCoV can also be divided into two biotypes, feline infectious peritonitis virus (FIP virus, FIPV; virulent FCoV) and feline enteric coronavirus (FECV; avirulent FCoV), according to its pathogenesis in animals [6]. FIPV infection typically causes a lethal disease in Felidae known as FIP [7]. The hallmark pathological findings of FIP in domestic cats are serous fluid in peritoneal and pleural cavities, pyogranulomatous lesions in internal organs, and disseminated fibrinous on the serosal and organ surfaces [6]. The FIPV-infected cells in the lesion and serous effusion predominantly consist of macrophages. FIPV-infected macrophages have been suggested to exacerbate the disease condition [8,9,10].

FCoVs attach to the cell surface through feline aminopeptidase N (fAPN) as the viral receptor. FCoVs enter the cell through endocytosis after attachment to the fAPN [6]. It is speculated that low endosomal pH triggers the conformation change of the spike protein of FCoV that releases the virus from the endosome to the cytosol. However, the timing of cytosol entry of each serotype was unclear.

FCoV infection is dependent on intracellular cholesterol [11]. Our previous study demonstrated that the triazole antifungal agent itraconazole blocks the intracellular trafficking of cholesterol and inhibits type I FCoV infection in a feline cell line (felis catus whole fetus-4 cells: fcwf-4 cells) [12]. Similar results were observed with U18666A, the widely studied cholesterol transport inhibitor [11]. Other researchers reported that itraconazole and U18666A blocked cholesterol egress from late endosomes [13,14,15]. Furthermore, cholesterol accumulation was reported to disrupt late endosome function [16,17]. Based on this, cholesterol transport inhibitors are hypothesized to inhibit the release of type I FCoV into the cytosol by indirectly disrupting the late endosome function. However, as described above, the site of endosomal release of both FCoV serotypes has not been investigated.

In this study, we demonstrated that type I FCoV cannot replicate in cells unless it reaches late endosomes, whereas type II FCoV can even though it does not reach late endosomes. Moreover, we investigated the effects of cholesterol-accumulating triazoles (itraconazole and posaconazole) and non-cholesterol-accumulating triazoles (voriconazole and fluconazole) [18] on the infectivity of FCoV in established feline cell lines (*Felis catus* whole fetus-4 cells (fcwf-4 cells)) and feline primary macrophages.

## 2. Results

### 2.1. Cytotoxicity of Compounds

**Figure 1:**
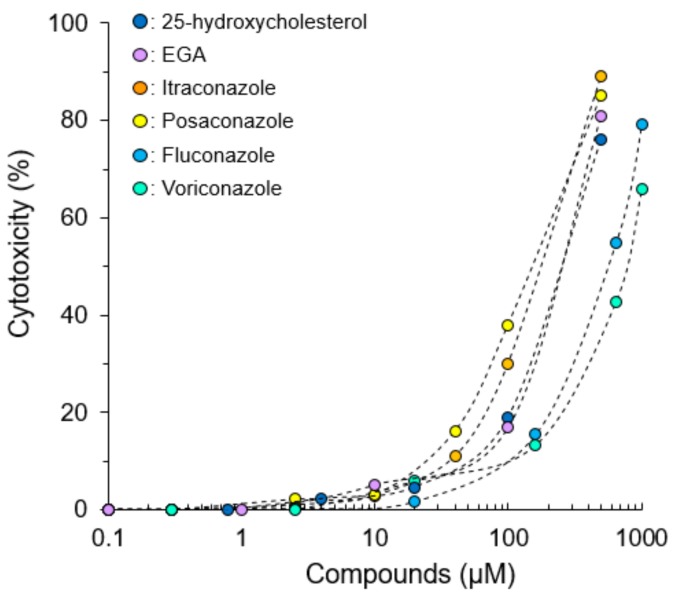
Cytotoxic effects of compounds on *Felis catus* whole fetus-4 cells (fcwf-4 cells). Data represent three independent experiments.

Cytotoxicity assay was performed to determine the non-toxic concentration of compounds against fcwf-4 cells (Figure 1). The concentrations used in this study were selected because they had low cytotoxic effects (cell viability: >83–90%) on feline cells.

### 2.2. The Effects of a Late Endosomal Trafficking Inhibitor on FCoV Infection

**Figure 2:**
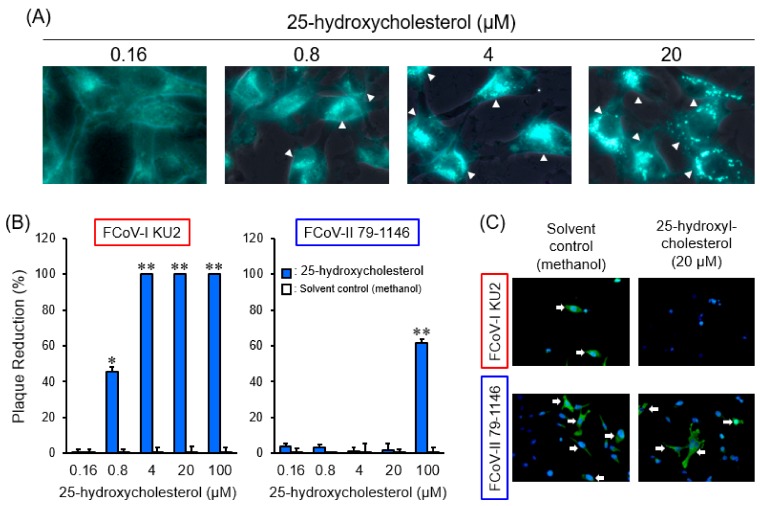
The effects of 25-hydroxycholesterol on feline coronavirus (FCoV) infection. (**A**) Accumulation of intracellular cholesterol in fcwf-4 cells pretreated with different concentrations of 25-hydroxycholesterol. The arrowheads point to the cells with accumulated intracellular cholesterol. (**B**) Plaque reduction in FCoV-infected fcwf-4 cells treated with different concentrations of 25-hydroxycholesterol. *, *p* < 0.05 vs. solvent control. **, *p* < 0.01 vs. each solvent control. Data represent four independent experiments. (**C**) Effects of 25-hydroxycholesterol on the viral protein. FCoV nucleocapsid (N) protein expression was evaluated by immunofluorescence assay (IFA). Nuclei were stained with 4′,6-diamidino-2-phenylindole (DAPI). The arrows point to the cells with expressing FCoV N protein.

**Figure 3:**
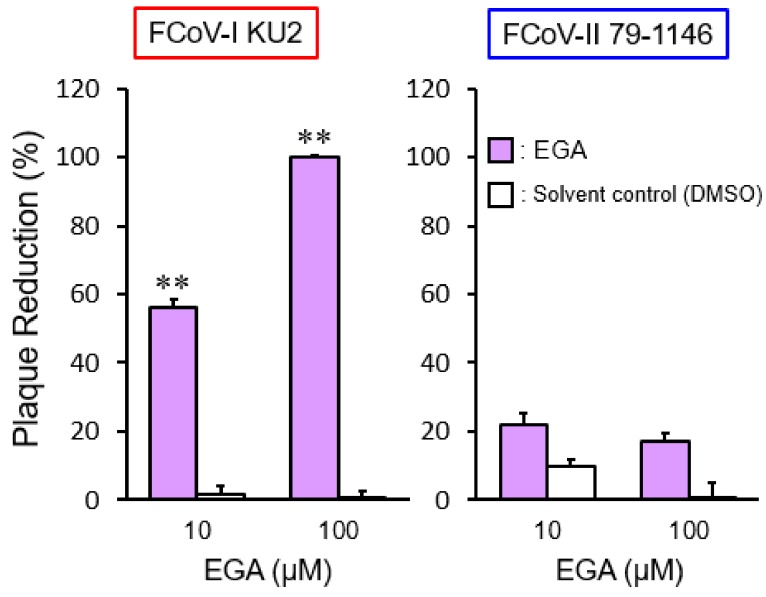
The effects of 4-bromobenzaldehyde N-(2,6-dimethylphenyl) semicarbazone (EGA), which specifically inhibits transport from early to late endosomes, on FCoV infection. **, *p* < 0.01 vs. each solvent control. Data represent four independent experiments.

To confirm whether FCoV accesses late endosomes for cell entry, we investigated the effects of 25-hydroxycholesterol (25-HC), a non-specific late endosome trafficking inhibitor [19], on FCoV. We assessed whether 25-HC inhibits late endosome function using intracellular cholesterol accumulation as an index. The 25-HC affected cholesterol accumulation in a dose-dependent manner (Figure 2A). We next examined whether 25-HC inhibits FCoV infection. The 25-HC exhibited antiviral activity in a dose-dependent manner on the type I FCoV (FIPV) KU-2 strain (FCoV-I KU-2) infection (Figure 2B). Of note, the inhibitory effects of 25-HC on FCoV-I KU2 infection were correlated with the cholesterol accumulation level. On the other hand, 25-HC had no inhibitory effects on the type II FCoV (FIPV) WSU 79-1146 strain (FCoV-II 79-1146) infection, except for highly concentrated 25-HC (Figure 2B). immunofluorescence assay (IFA) was used to detect the expression of FCoV nucleocapsid (N) protein. The N protein levels of FCoV-I KU2 were reduced by 25-HC (Figure 2C), suggesting that type I FCoV enters via late endosomes. To confirm this hypothesis, we examined the effects of viral infection after 4-bromobenzaldehyde N-(2,6-dimethylphenyl) semicarbazone (EGA) treatment, which specifically inhibits transport from early to late endosomes [20]. EGA significantly suppressed FCoV-I KU2 infection, but not FCoV-II 79-1146 infection (Figure 3).

### 2.3. The Effects of Late Endosomal Function on FCoV in Fcwf-4 Cells

**Figure 4:**
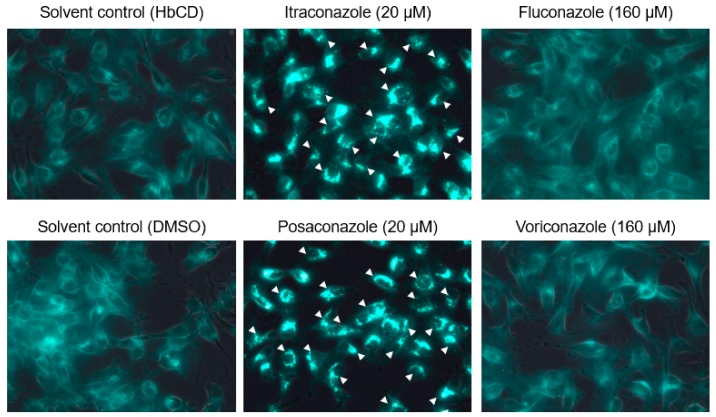
Accumulation of intracellular cholesterol in fcwf-4 cells pretreated with triazole antifungals. The arrowheads point to the cells with accumulated intracellular cholesterol.

**Figure 5:**
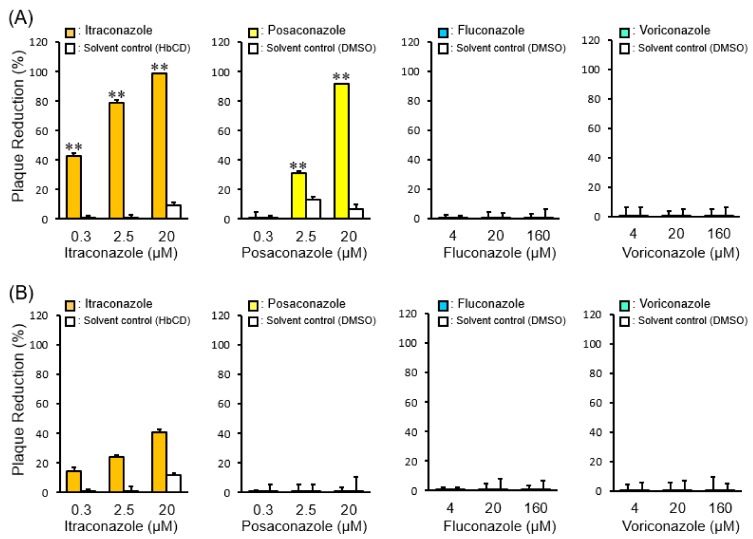
The effects of triazole antifungals on FCoV infection. (**A**) Plaque reduction in type I FCoV-infected fcwf-4 cells treated with different concentrations of triazole antifungals. **, *p* < 0.01 vs. each solvent control. Data represent four independent experiments. (**B**) Plaque reduction in type II FCoV-infected fcwf-4 cells treated with different concentrations of triazole antifungals. Data represent four independent experiments.

**Figure 6:**
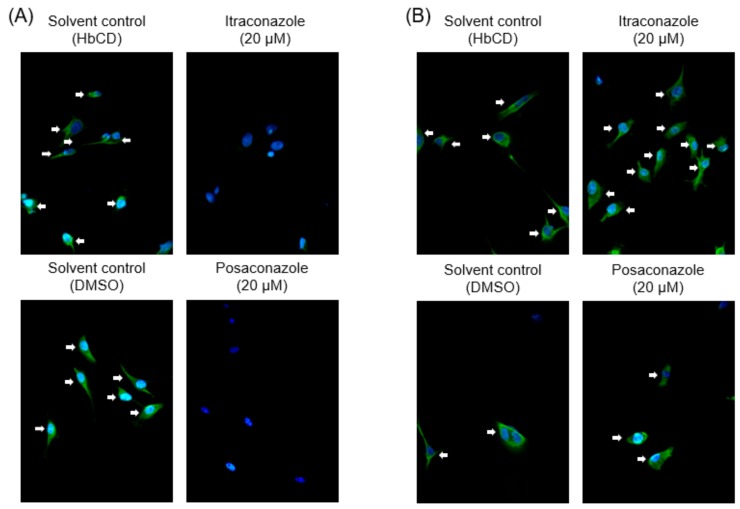
The effects of itraconazole and posaconazole on FCoV N protein expression in fcwf-4 cells. FCoV N protein expression was evaluated by IFA. Nuclei were stained with DAPI. (**A**) N protein expression of feline infectious peritonitis virus (FIPV)-I KU2-infected fcwf-4 cells. (**B**) N protein expression of FIPV-II 79-1146-infected fcwf-4 cells. The arrows point to the cells with expressing FCoV N protein.

Based on the above results and our previous study [11,12], different FCoV serotypes may enter feline cells through different endocytic routes: FCoV-I KU2 is released from late endosomes into the cytosol, whereas FCoV-II 79-1146 is released from early endosomes into the cytosol. Cholesterol accumulation was previously reported to disrupt late endosome function [16,17]. Therefore, we investigated the effects of cholesterol-accumulating triazoles (itraconazole and posaconazole) and non-cholesterol-accumulating triazoles (voriconazole and fluconazole) on the infectivity of FCoV. Similar to our previous studies, itraconazole caused cholesterol accumulation and inhibited FCoV-I KU2 infection in fcwf-4 cells (Figure 3 and Figure 4A). Similar effects on cholesterol accumulation and anti- viral activity have been observed with posaconazole (Figure 4 and Figure 5A). However, voriconazole and fluconazole did not cause cholesterol accumulation but still inhibited FCoV-I KU2 infection (Figure 4 and Figure 5A). IFA was used to detect the expression of FCoV N protein. The N protein levels of FCoV-I KU2 were reduced by itraconazole and posaconazole (Figure 6A). However, these triazoles did not inhibit FCoV-II 79-1146 infection (Figure 5B and Figure 6B). Thus, cholesterol accumulation has no influence on the efficiency of FCoV-II 79-1146 infection.

### 2.4. The Effects of Late Endosomal Function on FCoV in Primary Macrophages

**Figure 7:**
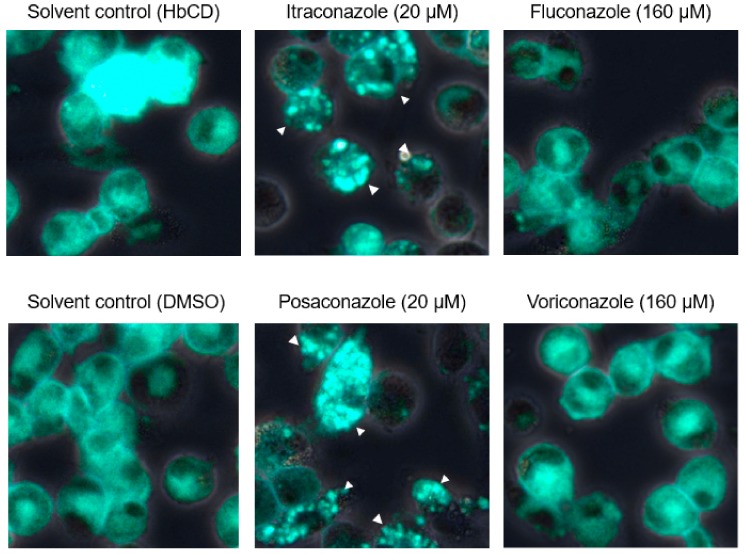
Accumulation of intracellular cholesterol in feline primary macrophages pretreated with triazole antifungals. The arrowheads point to the cells with accumulated intracellular cholesterol.

**Figure 8:**
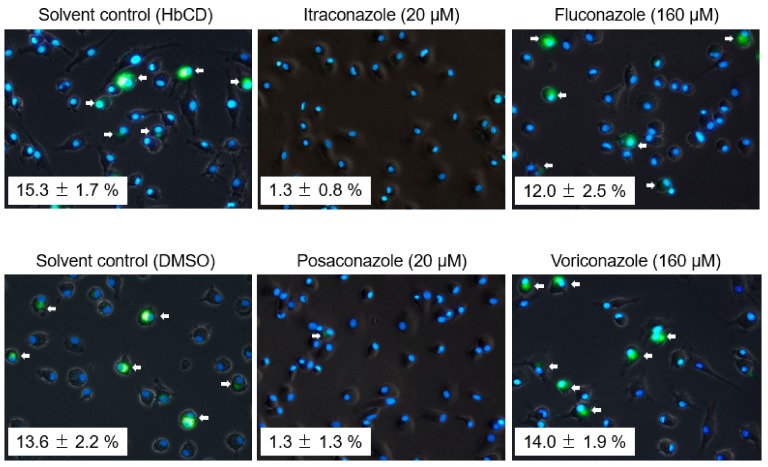
The effects of triazole antifungals on FCoV N protein expression in feline primary macrophages. FCoV N protein expression was evaluated by IFA. Nuclei were stained with DAPI. The value indicates the percentage of FCoV N-positive cells (mean ± SE) from four independent experiments (n = 3 cats). The arrows point to the cells with expressing FCoV N protein.

We next investigated whether findings similar to those observed in fcwf-4 cells are observed in macrophages, the target of virulent FCoV (FIPV). The concentration of triazole antifungals disrupting late endosome function in feline primary macrophages was confirmed, i.e., the concentration at which triazole antifungals accumulate intracellular cholesterol was measured. Both itraconazole and posaconazole clearly induced cholesterol accumulation at 20 μM (Figure 7). In contrast, voriconazole and posaconazole did not induce cholesterol accumulation even at the concentration limit not exhibiting cytotoxicity (Figure 7). We then examined whether FCoV can replicate in primary macrophages with disrupted late endosome function. In this experiment, only type I FCoV was used in consideration of the limited number of cats. In addition, no cytopathic effects are observed, and no virus is produced in the culture supernatant in type I FCoV-infected primary macrophages. Taking these conditions into consideration, viral infection of macrophages was judged based on the detection of FCoV N protein. As a result, N protein levels of FCoV-I KU2 were reduced by itraconazole and posaconazole, but not by voriconazole or fluconazole, in primary macrophages, similar to in fcwf-4 cells (Figure 8).

## 3. Discussion

**Figure 9:**
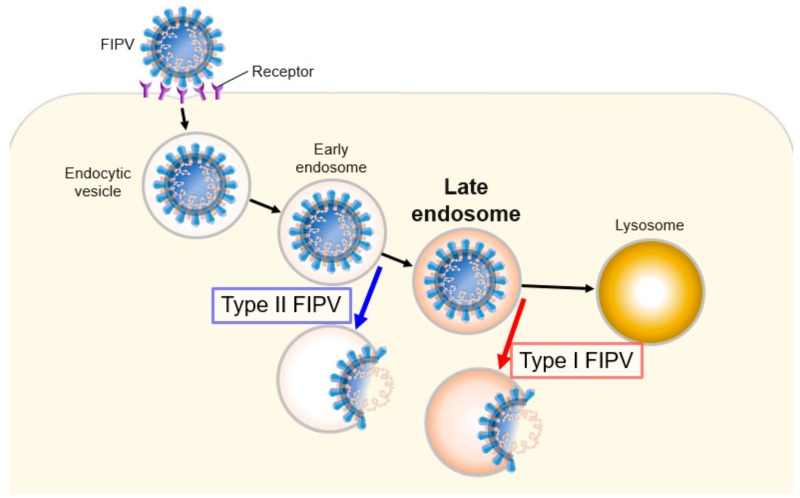
Schematic representation of FCoV entry through different endosomal compartments.

Viruses employ several entry strategies. After endocytosis, virus entry occurs at early or late endosomes [21,22,23,24,25]. According to previous studies, there are two cell entry types in coronavirus infection: Cytosol entry through early endosomes (e.g., MERS-CoV, infectious bronchitis virus, and human coronavirus NL-63) and late endosomes (e.g., SARS-CoV) [26,27]. Burkard et al. reported that FCoV (serotype is unclear) enters the cytosol through late endosomes [26]. We demonstrated that types I and II FCoV enter the cytosol through late and early endosomes, respectively (Figure 9).

To our knowledge, viruses that enter the cytosol at different times among serotypes have not been reported other than FCoV observed in this study. The FCoV serotypes are distinguished based on the amino acid sequence of S protein [2], i.e., timing of the serotypes to transit to the cytosol is dependent on S protein. Indeed, cleavage of coronavirus S protein in endosomes plays an important role in cytosol entry [28,29,30]. Cathepsin L is an endosomal protease involved in S protein cleavage of coronaviruses [31,32,33]. Cholesterol accumulation in late endosomes is observed in cells with inhibited cathepsin L activity [34]. Of note, the inhibition of cathepsin L activity does not influence the multiplication of FCoV-II 79-1146 [35]. Further investigation is necessary to clarify whether S protein cleavage by cathepsin L or cholesterol transport influences cytosol entry by FCoV.

The 25-hydroxycholesterol is oxysterol produced by cholesterol-25-hydroxylase induced by interferon [36]. Although 25-hydroxycholesterol inhibits several viral infections [36,37,38,39,40,41], many points remain unclear with regard to its antiviral activity. Civra et al. reported that 25-hydroxycholesterol accumulates cholesterol in late endosomes and inhibits rotavirus infection [19]. Based on their report, 25-hydroxycholesterol exhibits its antiviral activity through disrupting late endosome function. We confirmed that 25-hydroxycholesterol inhibits type I FCoV infection at the cholesterol-accumulating concentration, i.e., type I FCoV infection was blocked by 25-hydroxycholesterol through the same mechanism as that of rotavirus infection inhibition. Based on this finding, 25-hydroxycholesterol can be applied as a potent antiviral agent for FCoV. However, oxysterols, such as 25-hydroxycholesterol, induce arteriosclerosis [42,43]. Therefore, further investigation is necessary to use 25-hydroxycholesterol as an antiviral agent for FIP. High-concentration (100 μM) 25-hydroxycholesterol inhibited type II FCoV infection by 60%. The reason for this is unclear, but a large amount of 25-hydroxycholesterol incorporated by endocytosis may have physically blocked type II FCoV release from early endosomes.

Both FCoV serotypes use the endosomal pathway for cell entry [11,44]. However, the timing of cytosol entry of each serotype was unclear. EGA prevents transport from early endosomes to late endosomes [20]. Type II FCoV infection was not inhibited by EGA, demonstrating that type II FCoV enters the cytosol before it reaches late endosomes. In contrast, EGA inhibited type I FCoV infection. Thus, type I FCoV requires late endosomes to enter the cytosol. We tried to directly demonstrate that type I FCoV reaches late endosomes, i.e., we investigated colocalization of a late endosome marker, Rab7, and the virion, but we were unable to find a commercial anti-Rab7 antibody that reacts with feline Rab7. It is necessary to produce an antibody reacting with feline Rab7 and confirm the hypothesis described above.

Based on previous reports, triazole antifungals are classified into two types: cholesterol-accumulating triazoles (itraconazole and posaconazole) and non-cholesterol-accumulating triazoles (voriconazole and fluconazole) [18]. To our knowledge, the reason for these differences has not been clarified. These differences may be due to the structure of each drug. The structure of itraconazole is similar to that of posaconazole but different from those of voriconazole and fluconazole. Trinh et al. reported that itraconazole and posaconazole bind the sterol-sensing domain (SDD) of Niemann–Pick disease type C1 (NPC1) [45]. U18666A and 25-hydroxycholesterol, which induce cholesterol accumulation similarly to itraconazole, bind SDD [46,47]. Unlike these drugs, voriconazole and fluconazole may be unable to bind SDD. On the other hand, it was suggested that itraconazole, posaconazole, U18666A, and 25-hydroxycholesterol interfere with the binding of type I FCoV to NPC1, in addition to inducing cholesterol accumulation, by binding SDD [48]. However, in cells treated with SDD-binding cholesterol, type I FCoV infection increased. Thus, inhibition of type I FCoV infection by the above drugs is not related to binding of the virus to the SDD of NPC1.

FCoV-infected macrophages play an important role in the development of FIP [8,9,10]. We previously analyzed the mechanism of FCoV infection in feline primary macrophages [44]. Type I and type II FCoV are incorporated by endocytosis when they attach to the cell surface of macrophages, but it is unclear why FCoV that entered macrophages was not degraded. Considering these results, FCoV incorporated by macrophages may escape degradation by macrophage lysosomes by escaping into the cytosol from early/late endosomes.

In general, the infectivity of type I FCoV in feline cells is lower than that of type II FCoV. Recently, O’Brien et al. established fcwf-4 cells (fcwf-4 CU) that permit type I FCoV replication [49]. They noted lower interferon sensitivity as a factor enabling type I FCoV infection of this cell line [49,50]. As described above, 25-hydroxycholesterol inhibiting type I FCoV infection is induced in cells by interferon, suggesting that 25-hydroxycholesterol is not induced in fcwf-4 CU even though interferon is produced after infection. If this hypothesis is correct, the reason for increased infection by type I FCoV of fcwf-4 CU may be reduced 25-hydroxycholesterol production.

In this study, we indirectly demonstrated that type I FCoV cannot enter the cytosol unless it reaches late endosomes, whereas type II FCoV can even though it does not reach late endosomes. Our findings warrant further studies to elucidate the mechanism of cell entry by FCoV.

## 4. Materials and Methods

### 4.1. Cell Cultures, Animals, and Viruses

The fcwf-4 cells (kindly supplied by Dr. M. C. Horzinek of Utrecht University) were grown in Eagle’s minimum essential medium (MEM) containing 50% L-15 medium, 5% fetal calf serum (FCS), 100 U/mL of penicillin, and 100 μg /mL of streptomycin. For the primary macrophages, feline primary macrophages were selected. Feline alveolar macrophages were obtained from four specific-pathogen-free (SPF) cats aged 3–5 years by bronchoalveolar lavage with Hank’s balanced salt solution. Feline primary macrophages were maintained in D-MEM supplemented with 10% FCS, 100 U/mL of penicillin, and 100 μg/mL of streptomycin. SPF cats were maintained in a temperature-controlled isolated facility. The experiment using animals was approved by the President of Kitasato University through the judgment of the Institutional Animal Care and Use Committee of Kitasato University (18-050) and performed in accordance with the Guidelines for Animal Experiments of Kitasato University. Sample sizes were determined based on our previous study, and the minimum number of cats was used. The FCoV-I KU-2 was isolated in our laboratory. The FCoV-II 79-1146 was kindly provided by Dr. M. C. Horzinek of Utrecht University. These viruses were grown in fcwf-4 cells at 37 °C.

### 4.2. Compounds

The 25-HC (cholesterol transport inhibitor), EGA (late endosome trafficking inhibitor), posaconazole, voriconazole, and fluconazole were purchased from Sigma Aldrich Japan (Tokyo, Japan). Itraconazole was purchased from Janssen Pharma-central K. K. (Tokyo, Japan). The 25-HC was dissolved in methanol. EGA, Posaconazole, voriconazole, and fluconazole were dissolved in dimethyl sulfoxide (DMSO). Itraconazole was dissolved in 40% (*w*/*v*) hydroxypropyl-beta-cyclodextrin (HbCD) solution. All compounds were adjusted to 10 mM with solvent, aliquoted, and stored at-30 °C until use. On the day of the experiments, these compounds were diluted to the desired concentrations in maintenance medium.

### 4.3. Cytotoxic Effects of Compounds

The fcwf-4 cells were seeded on 96-well plates. The compounds were added to the wells in triplicate. After incubation for defined periods, the culture supernatants were removed, WST-8 solution (Kishida Chemical, Osaka, Japan) was added, and the cells were returned to the incubator for 1 h. The absorbance of formazan produced was measured at 450 nm using a 96-well spectrophotometric plate reader, as described by the manufacturer. Percentage viability was calculated using the following formula: Cytotoxicity (%) = [(OD of compound-untreated cells−compound-treated cells)/(OD of compound-untreated cells)] × 100.

### 4.4. Virus Infection and Compound Treatment

Confluent fcwf-4 cell monolayers were cultured in medium containing compounds at the indicated concentrations in 24-well multi-plates at 37 °C for 24 h. Cells were washed and the virus (MOI 0.01) was adsorbed into the cells at 37 °C for 1 h. After washing, cells were cultured in carboxymethyl cellulose (CMC)-MEM. The cell monolayers were incubated at 37 °C for 48 h, fixed and stained with 1% crystal violet solution containing 10% buffered formalin, and the resulting plaques were then counted. The plaque reduction percentage was calculated using the following formula: Plaque reduction percentage (%) = [(plaque number of compound-treated cells)/(plaque number of compound-untreated cells)] × 100.

### 4.5. Immunofluorescence Assay

Feline cells (fcwf-4 cells or feline primary macrophages) were cultured in each well of an 8-well Lab-Tek Chamber Slide (Thermo Fisher Scientific, Waltham, MA, USA). Semi-confluent cell monolayers were cultured in medium containing each compound at 37 °C for 24 h. Cells were washed and the virus (MOI 0.01) was adsorbed into the cells at 37 °C for 1 h (3 h in the case of macrophages). After washing, cells were cultured in MEM. The cell monolayers were incubated at 37 °C. After 24 h, N protein levels were measured by immunofluorescence assay (IFA), as described previously [51]. To recognize FCoV N protein, mAb E22-2 (mouse IgG1) prepared by our laboratory was used [51]. Nuclei were stained with 4′,6-diamidino-2-phenylindole (DAPI; Dojindo Laboratories, Kumamoto, Japan).

### 4.6. Detection of Cellular Cholesterol

The cellular cholesterol content of fcwf-4 cells was evaluated by the Cholesterol Cell-Based Detection Assay Kit (Cayman chemical, USA) according to the manufacturer’s instructions. Briefly, fcwf-4 cells were grown on an 8-well Lab-Tek Chamber Slide (Thermo Fisher Scientific, Waltham, MA, USA). Semi-confluent fcwf-4 cell monolayers were cultured in medium containing compounds at 37 °C for 24 h. After fixing and staining, filipin III-binding cells were analyzed using a Leica DM4B microscope and LAS X integrated imaging system (Leica Microsystems, Wetzlar, Germany).

### 4.7. Statistical Analysis

Data from only two groups were analyzed by the Student’s *t*-test (Welch’s *t*-test or Bartlett’s test), and data from multiple groups were analyzed by one-way ANOVA followed by Tukey’s test.

## References

[1] Su S., Wong G., Shi W., Liu J., Lai A.C., Zhou J., Liu W., Bi Y., Gao G.F. (2016). Epidemiology, genetic recombination, and pathogenesis of coronaviruses. Trends Microbiol..

[2] Motokawa K., Hohdatsu T., Aizawa C., Koyama H., Hashimoto H. (1995). Molecular cloning and sequence determination of the peplomer protein gene of feline infectious peritonitis virus type I. Arch. Virol..

[3] Hohdatsu T., Okada S., Ishizuka Y., Yamada H., Koyama H. (1992). The prevalence of types I and II feline coronavirus infections in cats. J. Vet. Med. Sci..

[4] Kummrow M., Meli M.L., Haessig M., Goenczi E., Poland A., Pedersen N.C., Hofmann-Lehmann R., Lutz H. (2005). Feline coronavirus serotypes 1 and 2: Seroprevalence and association with disease in Switzerland. Clin. Diagn. Lab. Immunol..

[5] Wang Y.T., Chueh L.L., Wan C.H. (2014). An eight-year epidemiologic study based on baculovirus-expressed type-specific spike proteins for the differentiation of type I and II feline coronavirus infections. BMC Vet. Res..

[6] Tekes G., Thiel H.J. (2016). Feline coronaviruses: Pathogenesis of feline infectious peritonitis. Adv. Virus Res..

[7] Pedersen N.C. (2016). An update on feline infectious peritonitis: Virology and immunopathogenesis. Vet. J..

[8] Stoddart C.A., Scott F.W. (1989). Intrinsic resistance of feline peritoneal macrophages to coronavirus infection correlates with in vivo virulence. J. Virol..

[9] Takano T., Hohdatsu T., Toda A., Tanabe M., Koyama H. (2007). TNF-alpha, produced by feline infectious peritonitis virus (FIPV)-infected macrophages, upregulates expression of type II FIPV receptor feline aminopeptidase N in feline macrophages. Virology.

[10] Takano T., Ohyama T., Kokumoto A., Satoh R., Hohdatsu T. (2011). Vascular endothelial growth factor (VEGF), produced by feline infectious peritonitis (FIP) virus-infected monocytes and macrophages, induces vascular permeability and effusion in cats with FIP. Virus Res..

[11] Takano T., Endoh M., Fukatsu H., Sakurada H., Doki T., Hohdatsu T. (2017). The cholesterol transport inhibitor U18666A inhibits type I feline coronavirus infection. Antivir. Res..

[12] Takano T., Akiyama M., Doki T., Hohdatsu T. (2019). Antiviral activity of itraconazole against type I feline coronavirus infection. Vet. Res..

[13] Xu J., Dang Y., Ren Y.R., Liu J.O. (2010). Cholesterol trafficking is required for mTOR activation in endothelial cells. Proc. Natl. Acad. Sci. USA.

[14] Poh M.K., Shui G., Xie X., Shi P.Y., Wenk M.R., Gu F. (2012). U18666A, an intra-cellular cholesterol transport inhibitor, inhibits dengue virus entry and replication. Antivir. Res..

[15] Lyu J., Yang E.J., Shim J.S. (2019). Cholesterol trafficking: An emerging therapeutic target for angiogenesis and cancer. Cells.

[16] Amini-Bavil-Olyaee S., Choi Y.J., Lee J.H., Shi M., Huang I.C., Farzan M., Jung J.U. (2013). The antiviral effector IFITM3 disrupts intracellular cholesterol homeostasis to block viral entry. Cell Host Microbe.

[17] Sobo K., Le Blanc I., Luyet P.P., Fivaz M., Ferguson C., Parton R.G., Gruenberg J., van der Goot F.G. (2007). Late endosomal cholesterol accumulation leads to impaired intra-endosomal trafficking. PLoS ONE.

[18] Strating J.R., van der Linden L., Albulescu L., Bigay J., Arita M., Delang L., Leyssen P., van der Schaar H.M., Lanke K.H., Thibaut H.J. (2015). Itraconazole inhibits enterovirus replication by targeting the oxysterol-binding protein. Cell Rep..

[19] Civra A., Francese R., Gamba P., Testa G., Cagno V., Poli G., Lembo D. (2018). 25-Hydroxycholesterol and 27-hydroxycholesterol inhibit human rotavirus infection by sequestering viral particles into late endosomes. Redox Biol..

[20] Gillespie E.J., Ho C.L.C., Balaji K., Clemens D.L., Deng G., Wang Y.E., Elsaesser H.J., Tamilselvam B., Gaargi A., Dixon S.D. (2013). Selective inhibitor of endosomal trafficking pathways exploited by multiple toxins and viruses. Proc. Natl. Acad. Sci. USA.

[21] Vonderheit A., Helenius A. (2005). Rab7 associates with early endosomes to mediate sorting and transport of Semliki forest virus to late endosomes. PLoS Biol..

[22] Saeed M.F., Kolokoltsov A.A., Albrecht T., Davey R.A. (2010). Cellular entry of ebola virus involves uptake by a macropinocytosis-like mechanism and subsequent trafficking through early and late endosomes. PLoS Pathog..

[23] Gastaldelli M., Imelli N., Boucke K., Amstutz B., Meier O., Greber U.F. (2008). Infectious adenovirus type 2 transport through early but not late endosomes. Traffic.

[24] van der Meer Y., Snijder E.J., Dobbe J.C., Schleich S., Denison M.R., Spaan W.J., Locker J.K. (1999). Localization of mouse hepatitis virus nonstructural proteins and RNA synthesis indicates a role for late endosomes in viral replication. J. Virol..

[25] Quirin K., Eschli B., Scheu I., Poort L., Kartenbeck J., Helenius A. (2008). Lymphocytic choriomeningitis virus uses a novel endocytic pathway for infectious entry via late endosomes. Virology.

[26] Burkard C., Verheije M.H., Wicht O., van Kasteren S.I., van Kuppeveld F.J., Haagmans B.L., Pelkmans L., Rottier P.J., Bosch B.J., de Haan C.A. (2014). Coronavirus cell entry occurs through the endo-/lysosomal pathway in a proteolysis-dependent manner. PLoS Pathog..

[27] Stadler K., Ha H.R., Ciminale V., Spirli C., Saletti G., Schiavon M., Bruttomesso D., Bigler L., Follath F., Pettenazzo A. (2008). Amiodarone alters late endosomes and inhibits SARS coronavirus infection at a post-endosomal level. Am. J. Respir. Cell Mol. Biol..

[28] Belouzard S., Millet J.K., Licitra B.N., Whittaker G.R. (2012). Mechanisms of coronavirus cell entry mediated by the viral spike protein. Viruses.

[29] de Haan C.A., Stadler K., Godeke G.J., Bosch B.J., Rottier P.J. (2004). Cleavage inhibition of the murine coronavirus spike protein by a furin-like enzyme affects cell-cell but not virus-cell fusion. J. Virol..

[30] Simmons G., Reeves J.D., Rennekamp A.J., Amberg S.M., Piefer A.J., Bates P. (2004). Characterization of severe acute respiratory syndrome-associated coronavirus (SARS-CoV) spike glycoprotein-mediated viral entry. Proc. Natl. Acad. Sci. USA.

[31] Bosch B.J., Bartelink W., Rottier P.J. (2008). Cathepsin L functionally cleaves the severe acute respiratory syndrome coronavirus class I fusion protein upstream of rather than adjacent to the fusion peptide. J. Virol..

[32] Qian Z., Dominguez S.R., Holmes K.V. (2013). Role of the spike glycoprotein of human Middle East respiratory syndrome coronavirus (MERS-CoV) in virus entry and syncytia formation. PLoS ONE.

[33] Simmons G., Zmora P., Gierer S., Heurich A., Pöhlmann S. (2013). Proteolytic activation of the SARS-coronavirus spike protein: Cutting enzymes at the cutting edge of antiviral research. Antivir. Res..

[34] Cermak S., Kosicek M., Mladenovic-Djordjevic A., Smiljanic K., Kanazir S., Hecimovic S. (2016). Loss of Cathepsin B and l leads to lysosomal dysfunction, NPC-like cholesterol sequestration and accumulation of the key Alzheimer’s proteins. PLoS ONE.

[35] Regan A.D., Shraybman R., Cohen R.D., Whittaker G.R. (2008). Differential role for low pH and cathepsin-mediated cleavage of the viral spike protein during entry of serotype II feline coronaviruses. Vet. Microbiol..

[36] Liu S.Y., Aliyari R., Chikere K., Li G., Marsden M.D., Smith J.K., Pernet O., Guo H., Nusbaum R., Zack J.A. (2013). Interferon-inducible cholesterol-25-hydroxylase broadly inhibits viral entry by production of 25-hydroxycholesterol. Immunity.

[37] Li C., Deng Y.Q., Wang S., Ma F., Aliyari R., Huang X.Y., Zhang N.N., Watanabe M., Dong H.L., Liu P. (2017). 25-Hydroxycholesterol protects host against Zika virus infection and its associated microcephaly in a mouse model. Immunity.

[38] Civra A., Cagno V., Donalisio M., Biasi F., Leonarduzzi G., Poli G., Lembo D. (2014). Inhibition of pathogenic non-enveloped viruses by 25-hydroxycholesterol and 27-hydroxycholesterol. Sci. Rep..

[39] Shrivastava-Ranjan P., Bergeron É., Chakrabarti A.K., Albariño C.G., Flint M., Nichol S.T., Spiropoulou C.F. (2016). 25-Hydroxycholesterol inhibition of Lassa virus infection through aberrant GP1 glycosylation. MBio.

[40] Dong H., Zhou L., Ge X., Guo X., Han J., Yang H. (2018). Antiviral effect of 25-hydroxycholesterol against porcine reproductive and respiratory syndrome virus in vitro. Antivir. Ther..

[41] Shawli G.T., Adeyemi O.O., Stonehouse N.J., Herod M.R. (2019). The Oxysterol 25-Hydroxycholesterol Inhibits Replication of Murine Norovirus. Viruses.

[42] Kumar N., Singhal O.P. (1991). Cholesterol oxides and atherosclerosis: A review. J. Sci. Food Agric..

[43] Shibata N., Glass C.K. (2010). Macrophages, oxysterols and atherosclerosis. Circ. J..

[44] Takano T., Katada Y., Moritoh S., Ogasawara M., Satoh K., Satoh R., Tanabe M., Hohdatsu T. (2008). Analysis of the mechanism of antibody-dependent enhancement of feline infectious peritonitis virus infection: Aminopeptidase N is not important and a process of acidification of the endosome is necessary. J. Gen. Virol..

[45] Trinh M.N., Lu F., Li X., Das A., Liang Q., De Brabander J.K., Brown M.S., Goldstein J.L. (2017). Triazoles inhibit cholesterol export from lysosomes by binding to NPC1. Proc. Natl. Acad. Sci. USA.

[46] Kwon H.J., Abi-Mosleh L., Wang M.L., Deisenhofer J., Goldstein J.L., Brown M.S., Infante R.E. (2009). Structure of N-terminal domain of NPC1 reveals distinct subdomains for binding and transfer of cholesterol. Cell.

[47] Head S.A., Shi W.Q., Yang E.J., Nacev B.A., Hong S.Y., Pasunooti K.K., Li R.J., Shim J.S., Liu J.O. (2016). Simultaneous targeting of NPC1 and VDAC1 by itraconazole leads to synergistic inhibition of mTOR signaling and angiogenesis. ACS Chem. Biol..

[48] Takano T., Satomi Y., Oyama Y., Doki T., Hohdatsu T. (2016). Differential effect of cholesterol on type I and II feline coronavirus infection. Arch. Virol..

[49] O’Brien A., Mettelman R.C., Volk A., André N.M., Whittaker G.R., Baker S.C. (2018). Characterizing replication kinetics and plaque production of type I feline infectious peritonitis virus in three feline cell lines. Virology.

[50] Mettelman R.C., O’Brien A., Whittaker G.R., Baker S.C. (2019). Generating and evaluating type I interferon receptor-deficient and feline TMPRSS2-expressing cells for propagating serotype I feline infectious peritonitis virus. Virology.

[51] Hohdatsu T., Sasamoto S., Koyama H. (1991). Antigenic analysis of feline coronaviruses with monoclonal antibodies (MAbs): Preparation of MAbs which discriminate between FIPV strain 79-1146 and FECV strain 79-1683. Vet. Microbiol..

